# Safety of primaquine in infants with *Plasmodium vivax* malaria in Papua, Indonesia

**DOI:** 10.1186/s12936-019-2745-7

**Published:** 2019-04-02

**Authors:** Agus Setyadi, Eggi Arguni, Enny Kenangalem, Afdhal Hasanuddin, Daniel A. Lampah, Kamala Thriemer, Nicholas M. Anstey, Paulus Sugiarto, Julie A. Simpson, Ric N. Price, Nicholas M. Douglas, Jeanne R. Poespoprodjo

**Affiliations:** 1 Department of Child Health, Faculty of Medicine, Public Health and Nursing, Universitas Gadjah Mada, Yogyakarta, Indonesia; 2 Timika Malaria Research Programme, Papuan Health and Community Development Foundation, Timika, Papua Indonesia; 3 Mimika District Hospital, Timika, Papua Indonesia; 4 Mitra Masyarakat Hospital, Timika, Indonesia; 5 Centre for Epidemiology and Biostatistics, Melbourne School of Population and Global Health, University of Melbourne, Melbourne, Australia; 6 Global Health Division, Menzies School of Health Research and Charles Darwin University, Darwin, NT Australia; 7 Division of Medicine, Royal Darwin Hospital, Darwin, NT Australia; 8 Centre for Tropical Medicine, Nuffield Department of Clinical Medicine, University of Oxford, Oxford, UK; 9 Mahidol-Oxford Tropical Medicine Research Unit (MORU), Faculty of Tropical Medicine, Mahidol University, Bangkok, Thailand

**Keywords:** *Plasmodium vivax*, Infants, Primaquine, Safety, Evaluation

## Abstract

**Background:**

Primaquine (PQ) prevents relapses of vivax malaria but may induce severe haemolysis in glucose-6-phosphate dehydrogenase (G6PD) deficient patients. Data on the safety of primaquine in infants are limited.

**Methods:**

A retrospective, hospital-based cohort study of infants aged 1–12 months with vivax malaria was carried out in Timika, Papua province, Indonesia. Risks of admission, death and severe haematological outcomes within 30 days of first presentation were compared between infants who did and did not receive primaquine. Infants were not tested routinely for G6PD deficiency as per local guidelines.

**Results:**

Between 2004 and 2013, 4078 infants presented to the hospital for the first time with vivax malaria, of whom 3681 (90.3%) had data available for analysis. In total 1228 (33.4%) infants were aged between 1 and 6 months and 2453 (66.6%) between 6 and 12 months of age. Thirty-three (0.9%) patients received low-dose primaquine (LDP), 174 (4.7%) received high-dose primaquine (HDP), 3432 (93.2%) received no primaquine (NPQ) and 42 patients received either a single dose or an unknown dose of primaquine. The risk of the Hb concentration falling by > 25% to less than 5 g/dL was similar in the LDP or HDP groups (4.3%, 1/23) versus the NPQ group (3.5%, 16/461). Three infants (1.4%) died following receipt of PQ, all of whom had major comorbidities. Seventeen patients (0.5%) died in the NPQ group. None of the infants had documented massive haemolysis or renal impairment.

**Conclusions:**

Severe clinical outcomes amongst infants treated with primaquine in Papua were rare. The risks of using primaquine in infancy must be weighed against the risks of recurrent vivax malaria in early life.

## Background

There are almost 12 million cases of *Plasmodium vivax* malaria each year [1]. The greatest burden of these infections is in poorly resourced communities where the disease is associated with severe anaemia and other adverse health and developmental effects in young children [2–6]. Much of the morbidity and transmission potential of *P. vivax* infections is attributable to relapsing disease arising from dormant liver stages of the parasite (hypnozoites) [7, 8].

The only widely available hypnozoitocidal agent for radical cure of *P. vivax* infection is primaquine (PQ), an 8-aminoquinoline anti-malarial drug [9, 10] which has potential to reduce the adverse consequences of severe anaemia due to recurrent episodes of parasite induced haemolysis [11]. This is particularly important in equatorial regions where the risk of *P. vivax* relapse is high and the interval between relapses is short [12, 13].

Primaquine results in oxidant stress which can cause severe and potentially fatal, drug-induced haemolysis in glucose-6-phosphate dehydrogenase (G6PD) deficient individuals [14, 15]. G6PD deficiency is common in malaria endemic populations, reaching a prevalence of 40% in some regions [16]. The overall risk–benefit of primaquine therapy for vivax malaria, therefore, hinges upon the dangers of drug induced haemolysis weighed against the dangers of recurrent parasite induced haemolysis and other malaria-related morbidities.

In 2015, the World Health Organization (WHO) anti-malarial guidelines reduced the lower age threshold for recommending the use of primaquine from 4 years of age to those over 6 months old, with close monitoring for signs of haemolysis [10]. Although the WHO recommends that patients be tested for G6PD deficiency prior to administration of primaquine, in reality, G6PD testing is rarely available in poorly resourced endemic regions [17–19].

This study examined the safety outcomes of pragmatic and discretionary use of primaquine in infants at Mitra Masyarakat Hospital in southern Papua, an area where the risk of severe anaemia associated with *P. vivax* relapse is high and the prevalence of G6PD deficiency is low [2].

## Methods

### Study site

Timika (Papua, Indonesia) is an area of unstable malaria transmission, with little seasonal variation in the climate (Mimika Regency Statistics, 2013). The population in 2012 was estimated to be 202,350 with the majority living in the lowland regions. The annual estimated incidence of malaria in the area has declined from 876 per 1000 population in 2004 [20] to 450 per 1000 in 2013 (Annual Health Report, Mimika District-2013) with approximately equal proportions of *P. vivax* and *Plasmodium falciparum* infections. The local *P. vivax* strains are highly resistant to chloroquine and have a relapse interval of 3–4 weeks [12, 13]. About half of the population own insecticide-treated bed nets [21].

Mitra Masyarakat Hospital (RSMM) was the only hospital in the region until November 2008, when the second hospital started to operate. Since 2009 RSMM has received about 80% of patients presenting to hospital with malaria [22]. RSMM has a functioning high care unit for critically ill infants and children.

### Study population

The ethnic groups in Timika are categorized into highland and lowland Papuans and non-Papuan Indonesians. Diarrhoea, lower respiratory tract infections and malaria are the predominant causes of morbidity and mortality in young children. The infant mortality rate is approximately 54 per 1000 live births [23] and the prevalence of G6PD deficiency is 1.3% (Pava et al. pers. commun.).

### Study design and data sources

This was a retrospective cohort study using routinely collected surveillance data gathered at RSMM hospital between April 2004 and December 2013. Clinical and demographic details (including the date of presentation, age, sex, ethnicity, and clinical diagnoses [International Classification of Diseases-ICD 10]) assigned by the attending physician) of every patient presentation to hospital were recorded in a Q-Pro™ database. Each patient was identified by a unique hospital record number (HRN). Electronic data were also available from laboratory and pharmacy records and were merged with the demographic and clinical dataset using the HRN and date. For the purposes of this analysis, the merged database was limited to infants aged between 1 and 12 months presenting for the first time with *P. vivax* mono- or mixed *Plasmodium* species infection. Healthcare at the RSMM hospital is free to the majority of the local population and since the hospital has better facilities and clinicians are available 24 h a day, most patients prefer to attend the hospital to primary care facilities.

Hospital protocol dictates that any patient presenting with fever or history of fever or any patient who is severely ill should be checked for malaria by microscopy using Giemsa stained thick blood smears. Thin blood smears were performed if the parasitaemia was too high to count by thick film examination. The hospital microscopists received refresher training annually. Testing for G6PD deficiency was not available during the study period. Clinicians requested a complete blood count only when clinically indicated and this was done using a Coulter counter.

Prior to 2006, the first-line schizontocidal treatment for uncomplicated malaria in infants due to any *Plasmodium* species was 7 days of quinine. After March 2006, infants weighing more than 5 kgs received dihydroartemisinin-piperaquine (DHA + PIP) [24].

### Definitions and outcomes of interest

Infants were defined as children aged between 1 and 12 months of age. Children less than 1 month old were excluded to avoid confounding from perinatal mortality. For infants with multiple presentations with *P. vivax* infection during the first year of life, only the first episode was included in this analysis. The total primaquine dose used was classified as no primaquine (NPQ), high dose primaquine (HDP; ≥ 5 mg/kg), low dose (LDP; ≥ 1.5 to < 5 mg/kg), unknown dose (matched primaquine prescription record but unable to determine dose) or single dose (< 1.5 mg/kg) [25].

The outcomes of interest were requirement for admission to hospital, all-cause death, severe anaemia (Haemoglobin concentration [Hb] < 5 g/dL) at any point during follow-up and a clinically significant drop in haemoglobin (reduction in Hb from baseline of > 25% to an absolute Hb concentration < 5 g/dL). Except in severe G6PD deficiency variants (such as “Mediterranean”), daily treatment with primaquine typically results in a nadir in haemoglobin around day 7 [17]. By this point, the rate of oxidative haemolysis has decreased due to the higher proportion of relatively G6PD replete young red blood cells and red cell production has increased to compensate sufficiently for the rate of loss. Outcomes, including death, occurring within the first 48 h were excluded as it was felt that these could not be caused plausibly by primaquine treatment. The timeline for possible primaquine associated haematological outcomes in this study was restricted to 3 to 30 days following initial presentation with vivax malaria.

### Statistical analysis

All statistical analyses were done in STATA^®^ version 15.1 (College Station, Texas). The risks of admission to hospital and death were presented visually using Kaplan–Meier failure curves. Normally distributed haemoglobin data were presented as mean and standard deviation. The risks of the binary safety outcomes of interest were presented as frequencies (percent) stratified by age group (1–6 months, 6–12 months), primaquine dose (NPQ, LDP, HDP) and, in the case of admission and death, period of follow-up (day 3–30, day 3–90). Comparisons of the frequency of safety outcomes were compared between those receiving no primaquine and those receiving either low dose or high dose primaquine and presented as odds ratios with 95% confidence intervals. Due to the very small numbers of events and multiple comparisons, p values are not presented.

### Ethical approval

Ethical approval for this study was obtained from the Medical and Health Research Ethics Committee, Faculty of Medicine, Gadjah Mada University, Yogyakarta, Indonesia (KE/FK/544/EC) and the ethics committee of Menzies School of Health Research, Darwin, Australia (HREC 10.1397).

## Results

Between April 2004 and December 2013, 4078 infants presented to RSMM for the first time with *P. vivax* infection of whom 74 (1.8%) were under 1 month of age and excluded from subsequent analyses. Antimalarial prescription data were available for 3691 (92.2%) of the remaining infants. Ten (0.27%) infants died within the first 48 h of presentation and were censored prior to any of the clinical outcomes.

**Fig. 1:**
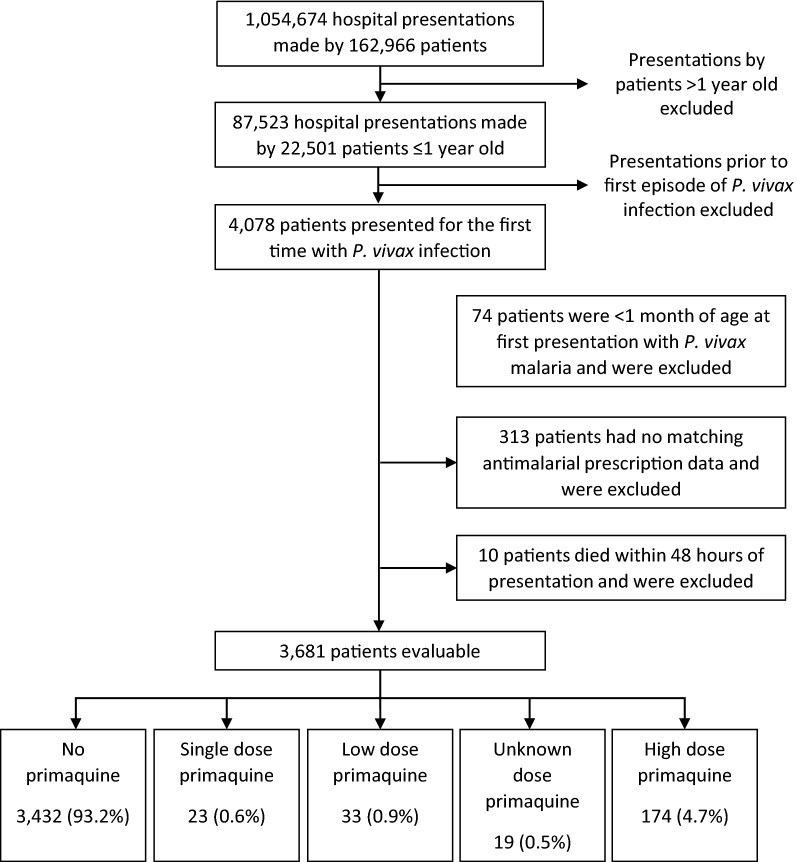
Study profile

**Fig. 2:**
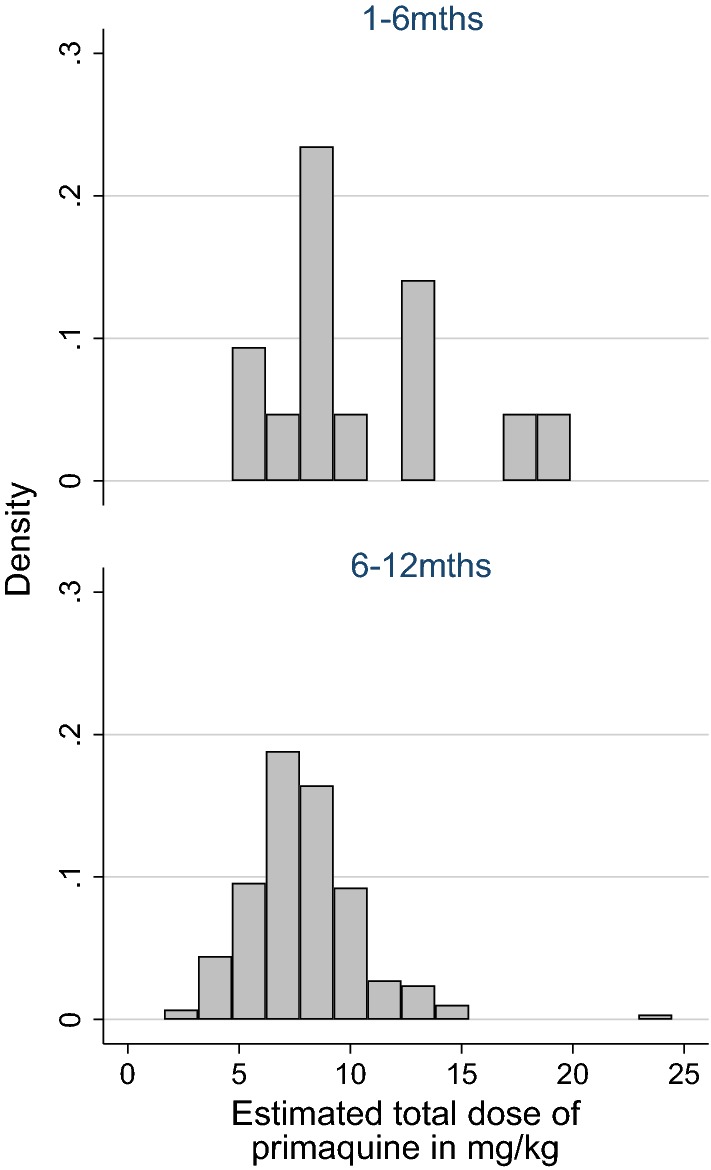
Histogram of estimated total primaquine dose in mg/kg by age category (excludes outlier values)

**Fig. 3:**
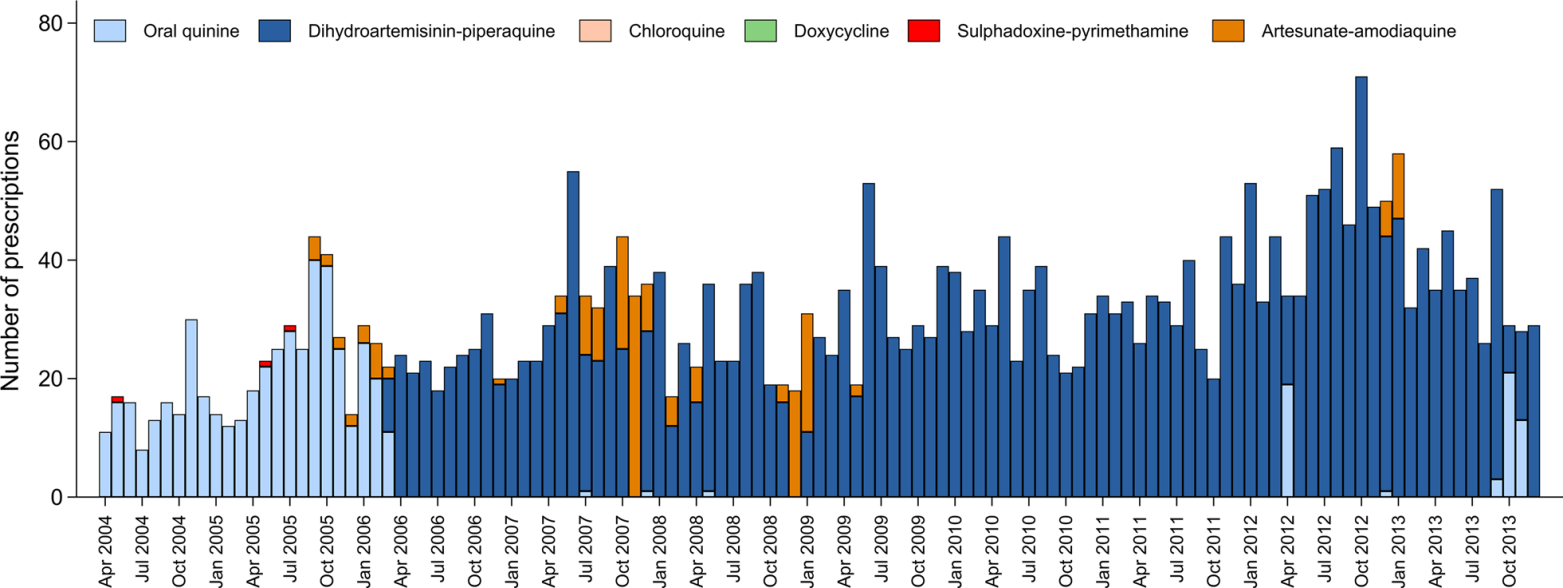
Oral schizontocidal prescriptions by month in patients ≤ 1 year old presenting with *P. vivax* monoinfection or mixed species infection

Of the 3432 infants in the analysis, 1208 (35.3%) were less than 6 months of age and 2224 (64.8%) between 6 and 12 months of age. Overall, 93.2% (3432/3681) of infants received no primaquine, 0.9% (33) received LDP, 4.7% (174) received HDP and 1.1% (42) received either a single dose or an unknown dose of primaquine (Table 1 and Fig. 1). The proportion of infants treated with primaquine was similar in outpatients compared with inpatients and those with mixed infections compared to those with *P. vivax* mono-infections. Infants aged 9–12 months were more likely to receive HDP (n = 137 (10.7%)) than younger babies (n = 37, 1.5%) (Table 1 and Fig. 2). Prior to the change of treatment policy in March 2006, oral quinine was the most commonly prescribed oral schizontocidal agent (n = 484, 89.5%) (Fig. 3). Thereafter, 91.9% (n = 2887) of infants received DHA + PIP.

**Table 1:** Demographic and clinical features of the infants included in the analyses

	Primaquine dose					
	No PQ	Single dose PQ	Low dose PQ	High dose PQ	Unknown dose	Total
	No. (%)	No. (%)	No. (%)	No. (%)	No. (%)	No. (%)
Age category						
0–3 m	464 (98.3)	2 (0.4)	0 (0)	6 (1.3)	0 (0)	472 (100)
3–6 m	744 (98.4)	1 (0.1)	2 (0.3)	7 (0.9)	2 (0.3)	756 (100)
6–9 m	1137 (97.3)	4 (0.3)	3 (0.3)	24 (2.1)	0 (0)	1168 (100)
9 m–1 yr	1087 (84.6)	16 (1.2)	28 (2.2)	137 (10.7)	17 (1.3)	1285 (100)
Gender						
Male	1831 (92.9)	12 (0.6)	16 (0.8)	100 (5.1)	11 (0.6)	1970 (100)
Female	1601 (93.6)	11 (0.6)	17 (1.0)	74 (4.3)	8 (0.5)	1711 (100)
Ethnicity						
Non Papuan	100 (90.1)	3 (2.7)	0 (0)	6 (5.4)	2 (1.8)	111 (100)
Highland Papuan	2804 (93.4)	17 (0.6)	27 (0.9)	142 (4.7)	13 (0.4)	3003 (100)
Lowland Papuan	528 (93.1)	3 (0.5)	6 (1.1)	26 (4.6)	4 (0.7)	567 (100)
Year						
2004	134 (82.7)	4 (2.5)	5 (3.1)	18 (11.1)	1 (0.6)	162 (100)
2005	265 (88.3)	10 (3.3)	15 (5.0)	8 (2.7)	2 (0.7)	300 (100)
2006	265 (90.1)	2 (0.7)	5 (1.7)	22 (7.5)	0 (0)	294 (100)
2007	393 (96.6)	1 (0.2)	1 (0.2)	11 (2.7)	1 (0.2)	407 (100)
2008	290 (90.1)	4 (1.2)	3 (0.9)	25 (7.8)	0 (0)	322 (100)
2009	358 (93.0)	1 (0.3)	1 (0.3)	23 (6.0)	2 (0.5)	385 (100)
2010	345 (91.5)	1 (0.3)	2 (0.5)	26 (6.9)	3 (0.8)	377 (100)
2011	377 (95.4)	0 (0)	1 (0.3)	10 (2.5)	7 (1.8)	395 (100)
2012	562 (95.6)	0 (0)	0 (0)	23 (3.9)	3 (0.5)	588 (100)
2013	443 (98.2)	0 (0)	0 (0)	8 (1.8)	0 (0)	451 (100)
Species						
Pv	2847 (93.3)	18 (0.6)	28 (0.9)	142 (4.7)	17 (0.6)	3052 (100)
Mix	585 (93.0)	5 (0.8)	5 (0.8)	32 (5.1)	2 (0.3)	629 (100)
Admission						
Outpatient	2598 (93.5)	10 (0.4)	19 (0.7)	140 (5.0)	13 (0.5)	2780 (100)
Inpatient	834 (92.6)	13 (1.4)	14 (1.6)	34 (3.8)	6 (0.7)	901 (100)
Baseline Hb	1939	17	23	87	7	2073
Mean (SD)	8.0 (2.2)	5.4 (2.1)	7.0 (2.1)	7.7 (2.2)	7.7 (3.4)	8.0 (2.2)
Total	3432 (93.2)	23 (0.6)	33 (0.9)	174 (4.7)	19 (0.5)	3681 (100)

*m* months, *yr* year, *Pv Plasmodium vivax*, *Mix* mixed *Plasmodium* species infection, *Hb* haemoglobin

### Haematological outcomes

At presentation haemoglobin concentration was available in 56.3% (2073/3681) of infants and at some point during the subsequent 3 to 30 days in 21.2% (782/3681) of infants. The mean haemoglobin concentration at presentation was 8.0 g/dL (SD = 2.2) in those not treated with primaquine, 7.0 g/dL (SD = 2.1) in those treated with LDP and 7.7 g/dL (SD = 2.2) in those treated with HDP. After excluding 234 infants with severe anaemia on presentation, a new diagnosis of severe anaemia occurred during follow-up in 4.4% (31/697) of patients who did not receive primaquine, 0% (0/6) of patients receiving LDP and 18.5% (5/27) of patients receiving HDP.

In 489 infants, a haemoglobin concentration was available both at presentation and between days 3 and 30. A clinically significant reduction in haemoglobin (reduction in Hb from baseline of > 25% to an absolute Hb concentration < 5 g/dL) was observed in 3.5% (16/461) of patients not treated with primaquine, 0% (0/4) of patients following LDP and 5.3% (1/19) of patients following HDP.

In total, 78.8% (2899/3681) of patients did not have a haemoglobin measurement between day 3 and 30 of follow-up. Since patients presenting with severe anaemia would most likely have clinical signs that would prompt a haemoglobin measurement, a sensitivity analysis was done in which patients without a follow-up haemoglobin measurement were assumed to not have severe anaemia. In this analysis the proportions of patients with new onset of severe anaemia during follow-up were 1.0% (31/3221) after NPQ, 0% (0/30) after LDP and 3% (5/165) after HDP and the proportions experiencing a clinically significant drop in haemoglobin were 0.5% (16/3432), 0% (0/33) and 0.6% (1/174), respectively.

### Readmission

**Fig. 4:**
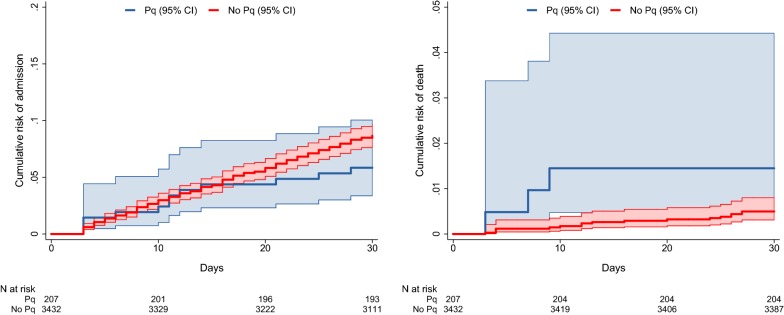
Cumulative risk of readmission and death by primaquine treatment category

A total of 1642 (44.6%) infants represented to hospital between 3 and 30 days following their initial presentation; 45.7% (1569/3432) of those not treated with primaquine, 36.4% (12/33) of those treated with LDP and 35.1% (61/174) of those treated with HDP. The risk of admission between day 3 and 30 in infants treated with either LDP or HDP was 5.8% (12/207) compared to 8.6% (296/3432) in those not treated with primaquine; Odds ratio (OR) = 0.65 (95% confidence interval [95% CI] 0.36–1.18) (Table 2 and Fig. 4).

**Table 2:** Frequency of outcomes of interest by primaquine dose category

	No PQ	LDP	HDP	LDP or HDP versus no PQ
	n/N (%)	n/N (%)	n/N (%)	OR (95%CI)
1–6 m				
Severe anaemia^a^	10/246 (4.1)	0/0 (–)	0/0 (–)	–
Hb fall > 25% to < 5 g/dL	4/183 (2.2)	0/0 (–)	0/0 (–)	–
Admission 3–30 d	118/1208 (9.8)	0/2 (0)	0/13 (0)	–
Death 3–30 d	11/1208 (0.91)	0/2 (0)	0/13 (0)	–
6–12 m				
Severe anaemia^a^	21/451 (4.7)	0/6 (0)	5/27 (18.5)	3.66 (1.28–10.4)
Hb fall > 25% to < 5 g/dL	12/278 (4.3)	0/4 (0)	1/19 (5.3)	1.01 (0.13–8.11)
Admission 3–30 d	178/2224 (8.0)	3/31 (9.7)	9/161 (5.6)	0.77 (0.42–1.40)
Death 3–30 d	6/2224 (0.27)	1/31 (3.2)	2/161 (1.2)	5.87 (1.46–23.6)
1–12 m				
Severe anaemia^a^	31/697 (4.5)	0/6 (0)	5/27 (18.5)	3.84 (1.39–10.6)
Hb fall > 25% to < 5 g/dL	16/461 (3.5)	0/4 (0)	1/19 (5.3)	1.26 (0.16–9.97)
Admission 3–30 d	296/3432 (8.6)	3/33 (9.1)	9/174 (5.2)	0.65 (0.36–1.18)
Death 3–30 d	17/3432 (0.50)	1/33 (3.0)	2/174 (1.2)	2.95 (0.86–10.2)

*PQ* primaquine, *LDP* low dose primaquine, *HDP* high dose primaquine, *n* number with outcome, *N* denominator, *OR* odds ratio, *95% CI* 95% confidence interval, *Hb* haemoglobin, *m* months, *d* day

^a^Limited to infants who were not severely anaemic at baseline

### Mortality

Twenty infants died between day 3 and day 30. The risk of death was 0.5% (17/3432) in infants not treated with primaquine, compared to 3.0% (1/33) in those treated with LDP and 1.1% (2/174) in those treated with HDP. Treatment with either LDP or HDP was associated with an OR of 2.95 (95% CI 0.86–10.2) for death. The clinical and haematological details of the patients who died are presented in Table 3. All infants had significant comorbidities other than *P. vivax* infection: 12 (60%) children had bacterial infection (bronchopneumonia or meningitis), 3 (15%) bronchopneumonia and gastroenteritis, 4 (20%) had tuberculosis, and 1 (5%) had gastroenteritis and malnutrition (Table 3). Four out of 17 infants who died having not been prescribed primaquine had their haemoglobin measured at both baseline and representation, two (50%) of whom had a clinically significant reduction. A 12-month old female died following HDP and had a clinically significant fall in haemoglobin from 6.4 to 4.6 g/dL, and an associated diagnosis of bronchopneumonia. A 12-month old male treated with HDP was diagnosed with chronic nephritic syndrome, tuberculosis and malnutrition and a 12-month old female treated with LDP had a diagnosis of malnutrition, dehydration and acidosis. None of the infants who died had a recorded diagnosis of blackwater fever or renal failure.

**Table 3:** Clinical Data of infants who died between day 3 and 30 of initial presentation

Patient number	Age	Sex	Ethnicity	Species	Initial Hb (g/dL)	Lowest F/U Hb (g/dL)	Days between presentation and lowest F/U Hb	Days between presentation and death	Schizontocidal drug used	Primaquine dose	Other clinical diagnoses assigned between presentation and death
1	1 m	M	HP	Pv	7.1	3.4	14	20	IV artesunate/DHA + PIP	Nil	Gastroenteritis Malnutrition Dehydration Pulmonary tuberculosis
2	1 m	M	HP	Mix	2.3	–	–	4	IV artesunate	Nil	Bacterial sepsis of newborn Neonatal jaundice Late metabolic acidosis of newborn Cerebral malaria
3	1 m	F	HP	Pv	–	9.3	27	27	DHA + PIP	Nil	Oral candidiasis Staphylococcal scalded skin syndrome Stevens-Johnsons syndrome Hypoglycaemia
4	2 m	M	HP	Pv	6.3	–	–	13	IV artesunate/DHA + PIP	Nil	Septic shock Respiratory distress syndrome of newborn Hypoalbuminaemia
5	2 m	F	HP	Mix	–	–	–	27	DHA + PIP	Nil	Bacterial meningitis Acute upper respiratory tract infection
6	2 m	M	HP	Pv	–	9.8	20	25	IV artesunate/DHA + PIP	Nil	Bronchopneumonia
7	4 m	M	HP	Pv	8.3	6.8	24	26	PO quinine	Nil	Gastroenteritis Acidosis Meningitis
8	4 m	F	HP	Pv	11.1	11.2	6	16	DHA + PIP	Nil	HIV Pulmonary tuberculosis Marasmus Candidiasis
9	4 m	M	HP	Pv	–	9.7	3	4	DHA + PIP	Nil	Bacterial meningitis
10	4 m	F	HP	Pv	–	8	20	26	DHA + PIP	Nil	Bronchopneumonia
11	5 m	M	HP	Pv	13	–	–	12	IV artesunate/DHA + PIP	Nil	Bronchopneumonia Septic shock
12	6 m	F	HP	Pv	3.5	–	–	4	IV/PO quinine	Nil	Bronchopneumonia Exfoliative dermatitis
13	6 m	F	HP	Mix	2.1	–	–	3	IV artesunate/DHA + PIP	Nil	Bronchopneumonia
14	8 m	F	HP	Pv	9.6	–	–	12	IV artesunate/DHA + PIP	Nil	Tuberculous meningitis Septic shock Conjunctivitis
15	10 m	M	NP	Pv	7.2	–	–	3	IV artesunate/DHA + PIP	High dose	Tuberculosis of skin and subcutaneous tissue
16	10 m	F	LP	Pv	–	–	–	10	DHA + PIP	Nil	Measles Gastroenteritis and colitis Bronchopneumonia
17	12 m	F	HP	Pv	6.4	4.6	3	9	PO quinine	High dose	Bronchopneumonia
18	12 m	M	HP	Pv	8.7	4.3	23	24	DHA + PIP	Nil	Gastroenteritis Dehydration Septic shock Acidosis Respiratory failure Febrile convulsion
19	12 m	F	HP	Pv	8.9	–	–	9	DHA + PIP	Nil	Pneumonia Septic shock
20	12 m	M	HP	Pv	7.9	–	–	7	DHA + PIP	Low dose	Gastroenteritis Malnutrition Dehydration Acidosis

*m* months, *M* male, *F* female, *HP* Highland Papuan, *LP* Lowland Papuan, *NP* non-Papuan, *Pv Plasmodium vivax*, *Mix* mixed *Plasmodium* species infection, *Hb* haemoglobin, *F/U* follow-up, *PO* oral, *IV* intravenous, *DHA *+ *PIP* dihydroartemisinin-piperaquine

## Discussion

In view of the risk of haemolysis, limited capacity of cardiorespiratory compensatory mechanisms and the difficulty diagnosing G6PD deficiency in young infants, the WHO does not recommend primaquine for infants under the age of 6 months [10, 26, 27]. However the risks of primaquine use in this age group need to be balanced against the significant risks of recurrent *P. vivax* infections in the first year of life, which in Papua-Indonesia, also includes severe anaemia and both direct and indirect mortality [2, 28]. Approximately 65% of children presenting for the first time with *P. vivax* infection to hospital in Timika will represent to hospital within 12 months, 50% will represent with malaria within that time period and 25% will require admission (about 40% of these admissions are attributable to malaria) (Patriani et al., pers.commun.).

At the local hospital in Timika, primaquine was prescribed in 6.8% (249/3681) of infants presenting with *P. vivax* infection. The proportion of infants requiring admission to the hospital within 3 to 30 days was lower in those treated with HDP (5.7%) compared to infants who did not receive primaquine (8.6%). A plausible explanation for this is that primaquine prevented vivax relapse and thus morbidity associated with recurrent malaria. Previous study has shown that the overall effectiveness of unsupervised primaquine at RSMM is poor, but that there is some benefit in young children, potentially because of parental influence increasing adherence to treatment [25]. Alternatively, these observations could reflect bias caused by purposeful selection of less vulnerable infants for primaquine treatment. However, given the similar proportions of inpatients and outpatients treated with primaquine the latter explanation seems unlikely.

Quantifying the acute hemolytic effect of primaquine in infants is confounded by the haemolytic effects of malaria itself along with any other concomitant morbidities [15, 28]. The most appropriate metric for clinically relevant haemolysis is disputed. Since the fractional fall in hemoglobin is correlated positively with the starting Hb, the metric chosen were both development of severe anaemia (Hb < 5 g/dL) and a composite measure of a high fractional fall (> 25%) to an absolute Hb below 5 g/dL, at which level the risk of mortality rises significantly [28].

The mean Hb concentration in infants at baseline was similar (7–8 g/dL) between treatment groups. The crude frequency of severe anaemia and a clinically significant drop in haemoglobin was higher amongst patients who received HDP (18.5% and 5.3% respectively) compared to those who did not receive primaquine (4.5% and 3.5% respectively) whereas neither of these adverse haematological outcomes occurred in patients treated with low dose primaquine. One infant who had a clinically significant drop in haemoglobin after HDP had an initial Hb level of 6.4 g/dL falling to 4.6 g/dL by day 3, and died 9 days after presentation with bronchopneumonia (Table 3). Since biochemical laboratory tests were not available in this infant, severe hemolysis with acute renal failure and associated pulmonary oedema could not be ruled out.

Profound haemolysis leading to severe anaemia, is likely to prompt representation to hospital and be detected clinically by the attending physician. Hence the incidence of severe anaemia amongst those who did not have a repeat haemoglobin was probably low. If this assumption is true, the true frequencies of new onset severe anaemia or a clinically significant reduction in haemoglobin following high dose primaquine are likely to be substantially lower than those observed (as demonstrated in the sensitivity analyses). Nevertheless, the frequency of severe haematological events at a population level needs to be considered in the context of the prevalence of the susceptible phenotype, namely severe G6PD deficiency, which in Timika is estimated to be about 1.3%.

Three infants treated with LDP or HDP died following treatment with primaquine. All three had significant comorbidity (tuberculosis in one case, severe malnutrition, gastroenteritis, dehydration and acidosis in another case and bronchopneumonia). The initial haemoglobin was less than 8 g/dL in all three infants, and thus a small fall in haemoglobin attributable to either acute malaria or drug, associated with significant co-morbidities, could have contributed to their clinical decline [29].

The relevance of these findings to other endemic locations needs to be considered with caution. In the study population the prevalence of G6PD deficiency (< 30% activity) is low and absent in those of highland ethnicity, who make up more than 70% of patients presenting to the hospital (Pava et al., pers.commun.). Elsewhere in Indonesia, the G6PD deficiency prevalence is much higher, the dominant variants being Vanua Lava, Viangchan and Chatam which are associated with enzyme activity of less than 10% [16, 19]. Use of primaquine without prior G6PD testing in those regions, particularly in infants, would carry significantly greater risk and G6PD testing would be mandatory prior to primaquine administration

This study has several important limitations. Firstly infants who died at home or visited other health facilities following their initial presentation to RSMM with vivax malaria will not have been detected. In a treatment seeking survey conducted in 2013, family members from households in which a member had died within the preceding year, reported that the death had occurred at the RSMM hospital in 30% of cases [30]. Hence whilst many deaths will have been identified by our surveillance, some would have been missed. This attrition bias is likely be similar for infants treated with or without primaquine. Secondly it was not possible to confirm that infants completed a full course of primaquine after discharge from the hospital, and a previous study showed poor effectiveness, which was assumed to be secondary to poor adherence to the 14-day primaquine regimen. Thus, incomplete treatment will have resulted in underestimation of the frequency of adverse events that would be observed in a fully supervised regimen [25]. Finally haemoglobin measures were not undertaken routinely. Most patients had their haemoglobin measured only once and this was generally on the day of admission or representation. Thereafter haemoglobin was only measured when there was clinical concern. In view of this, a composite measure of significant haemolysis was chosen, which comprised a high fractional fall to below an absolute concentration of 5 g/dL—a point at which anaemia would presumably have been apparent clinically, triggering laboratory investigation. The true minimum haemoglobin concentration for each individual may have been lower than the lowest measured concentration recorded in the hospital database.

## Conclusion

The clinical decision to give primaquine as radical treatment for *P. vivax* malaria and mixed infections in this vulnerable group without G6PD testing should be made with great caution, particularly in areas with a higher prevalence of G6PD deficiency. If primaquine is to be prescribed, parents and guardians must be informed of the early signs of haemolysis and advised to seek immediate medical attention should haemolysis occur [15, 31]. Considering the potential life-threatening consequences of haemolysis from either recurrent *P. vivax* malaria or antirelapse treatment in G6PD deficient infants, there is an urgent need for a highly sensitive point of care G6PD test to ensure that radical cure can be deployed safely to those who need it most.

## Abbreviations

PQ: primaquine
G6PD: glucose-6-phosphate dehydrogenase
RSMM: Mitra Masyarakat Hospital
HDP: high dose primaquine
LDP: low dose primaquine
NPQ: no primaquine
ICD: International Classification of Diseases
Hb: Haemoglobin
DHA + PIP: dihydroartemisinin-piperaquine
SD: standard deviation
STATA: statistics and data science
n: number with outcome
N: denominator
OR: odds ratio
95% CI: 95% confidence interval
m: months
yr: year
Pv: *Plasmodium vivax*
Mix: mixed *Plasmodium* species infection
M: male
F: female
HP: Highland Papuan
LP: Lowland Papuan
NP: non-Papuan
F/U: follow-up
PO: oral
IV: intravenous
